# Supplementary figures and images for: QTL Mapping for Wheat Seed Dormancy in a Yangmai16/Zhongmai895 Double Haploid Population
Source: Plants (Basel). 2023 Feb 8;12(4):759. doi: 10.3390/plants12040759 (PMC9967201; doi:10.3390/plants12040759)

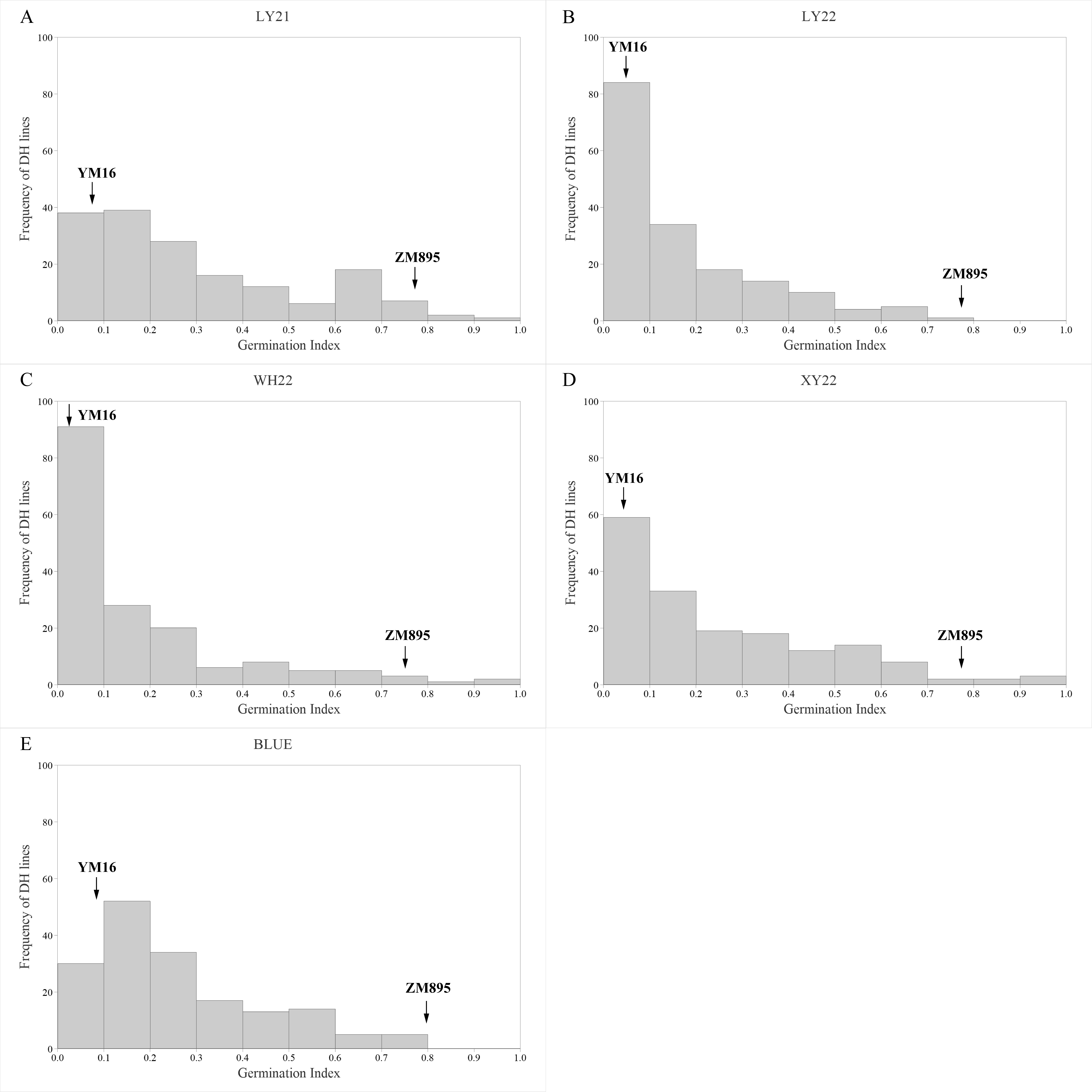

Supplement: Supplementary file 1 [file plants-12-00759-s001.zip › Figure S1.tif]

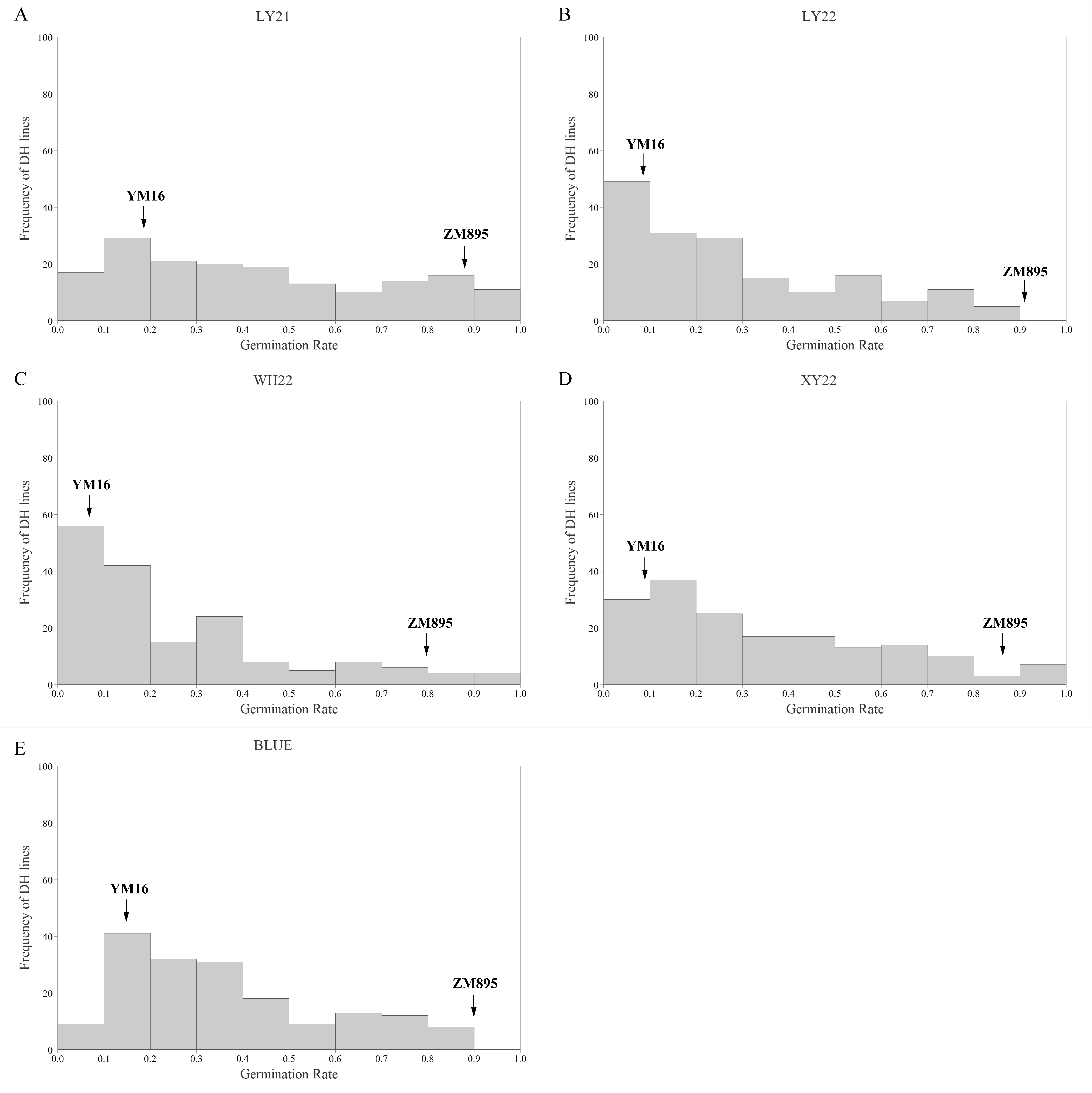

Supplement: Supplementary file 1 [file plants-12-00759-s001.zip › Figure S2.tif]

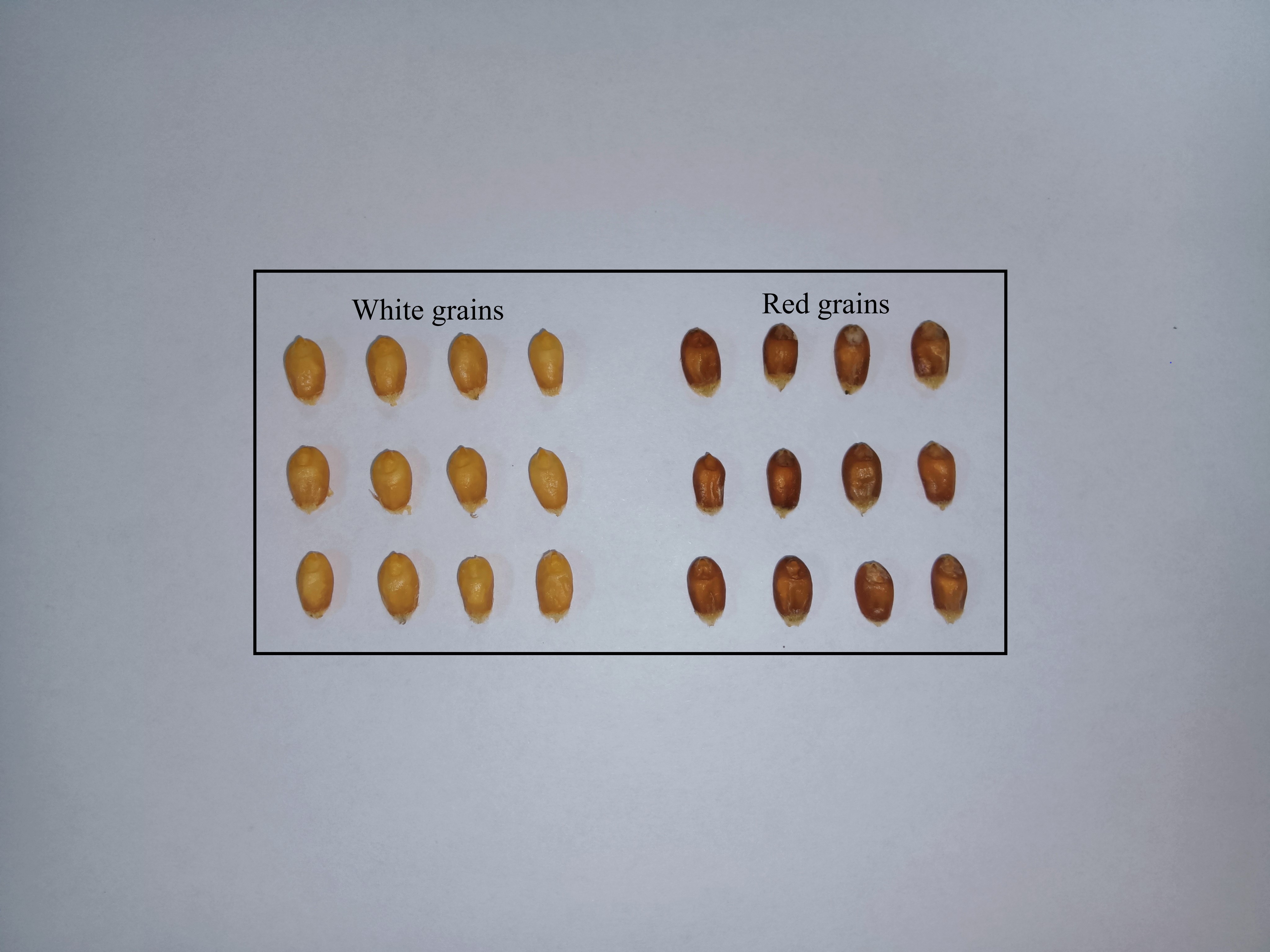

Supplement: Supplementary file 1 [file plants-12-00759-s001.zip › Figure S3.tif]

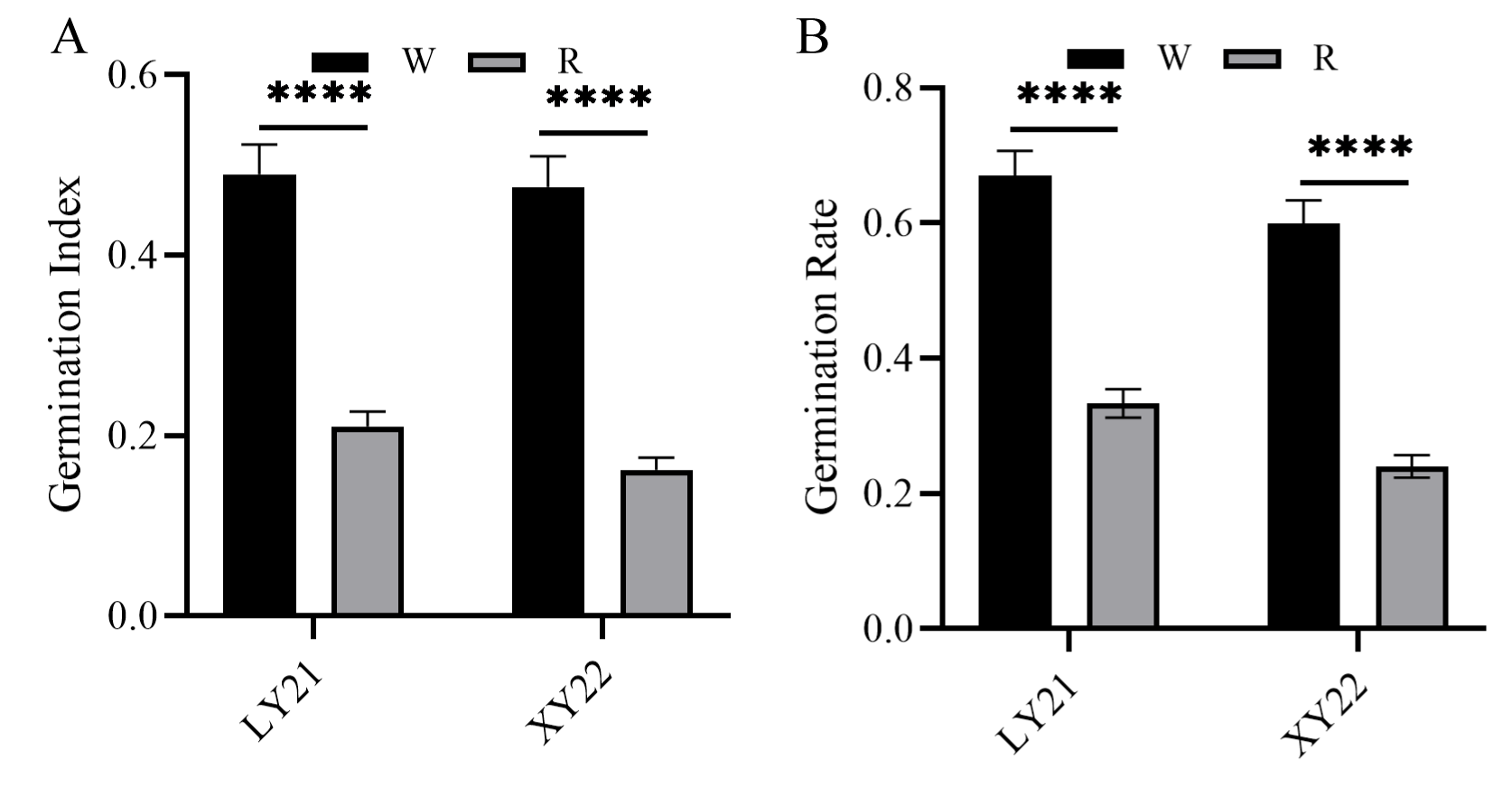

Supplement: Supplementary file 1 [file plants-12-00759-s001.zip › Figure S4.tif]

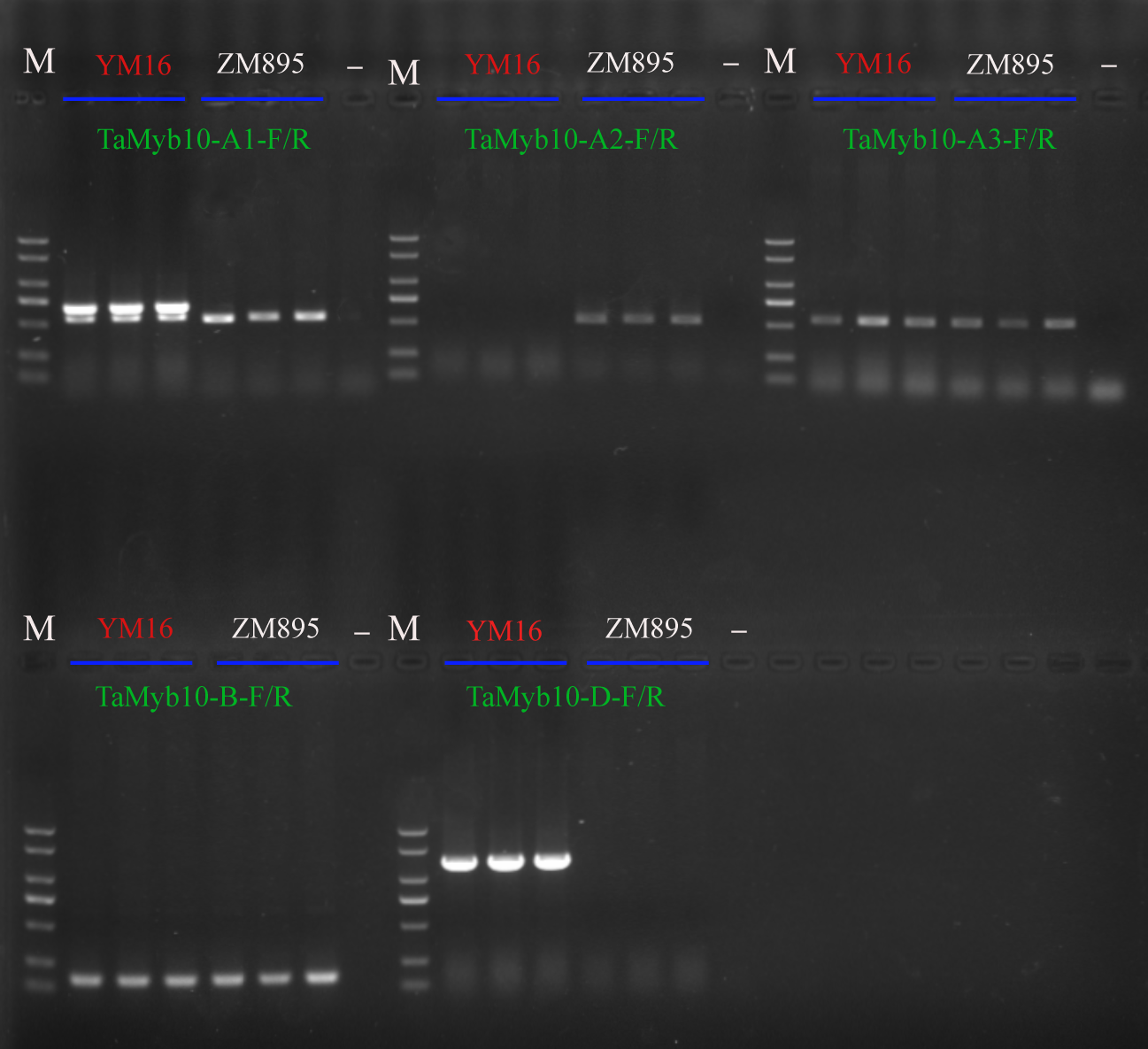

Supplement: Supplementary file 1 [file plants-12-00759-s001.zip › Figure S5.tif]

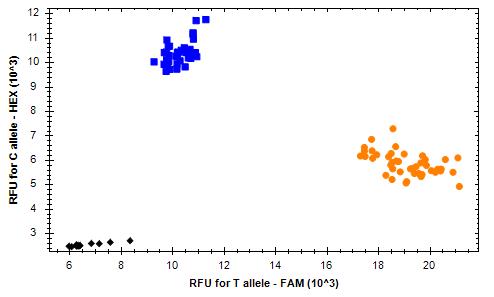

Supplement: Supplementary file 1 [file plants-12-00759-s001.zip › Figure S6.jpg]
